# Complete Genome Sequences of Four Extensively Drug-Resistant Acinetobacter baumannii Isolates from Thailand

**DOI:** 10.1128/MRA.00949-20

**Published:** 2020-10-01

**Authors:** Peechanika Chopjitt, Thidathip Wongsurawat, Piroon Jenjaroenpun, Parichart Boueroy, Rujirat Hatrongjit, Anusak Kerdsin

**Affiliations:** a Faculty of Public Health, Kasetsart University, Chalermphrakiat Sakon Nakhon Province Campus, Sakon Nakhon, Thailand; b Department of Biomedical Informatics, University of Arkansas for Medical Sciences, Little Rock, Arkansas, USA; c Division of Bioinformatics and Data Management for Research, Department of Research and Development, Faculty of Medicine Siriraj Hospital, Mahidol University, Bangkok, Thailand; d Faculty of Science and Engineering, Kasetsart University, Chalermphrakiat Sakon Nakhon Province Campus, Sakon Nakhon, Thailand; Portland State University

## Abstract

Here, we report the complete genome sequences of four clinical isolates of extensively drug-resistant Acinetobacter baumannii (XDRAB), isolated in Thailand. These results revealed multiple antimicrobial-resistant genes, each involving two sequence type 16 (ST16) isolates, ST2, and a novel sequence type isolate, ST1479.

## ANNOUNCEMENT

Acinetobacter baumannii is one of the multidrug-resistant bacteria listed as a priority by the World Health Organization, as it exhibits resistance to most commercially available antibiotics and causes hospital-acquired infections (1–3). In particular, carbapenem-resistant Acinetobacter baumannii (CRAB) infections present limited therapeutic options and are associated with high morbidity and mortality as well as longer hospitalization (3). Recently, incidences of extensively drug-resistant A. baumannii (XDRAB) infections have been reported worldwide, including in Thailand (4, 5). Four strains were isolated from individual sputum (*n* = 3) and bile (*n* = 1) samples by a tertiary hospital in northeastern Thailand between 2017 and 2018 (6). All isolates were cultured on sheep blood agar for DNA extraction. They were identified to the species level using *gyrB* multiplex PCR (7). These strains were resistant to 11 representative antimicrobial agents of all drug classes (piperacillin, piperacillin-tazobactam, ceftazidime, cefepime, imipenem, meropenem, ciprofloxacin, gentamicin, amikacin, trimethoprim-sulfamethoxazole, and tetracycline), whereas they had intermediate resistance to colistin, according to the Clinical and Laboratory Standards Institute (CLSI, 2020) guidelines (8). The four XDRAB strains were grouped into international clone II (*n* = 2) and no clonal group (*n* = 2) based on a multiplex PCR assay (9).

Bacterial genomic DNA samples extracted using ZymoBIOMICS DNA kits (Zymo Research, USA) were sequenced using the Oxford Nanopore Technologies (ONT) and Illumina platforms. Library preparation for ONT sequencing followed the rapid barcoding DNA sequencing protocol with the SQK-RBK004 kit without DNA size selection, to preserve the plasmid DNA, and the libraries were sequenced using a single R9.4.1/FLO-MIN106 flow cell on a MinION Mk1B sequencer. We base called and demultiplexed the raw data using Guppy v3.4.5 (ONT), specifying the high-accuracy model (-c dna_r9.4.1_450bps_hac.cfg). The ONT adapters were trimmed using Porechop v0.2.4 (https://github.com/rrwick/Porechop). Quality control of ONT reads was undertaken using NanoPlot v1.28.1 (https://github.com/wdecoster/NanoPlot). For the Illumina platform, the sequencing library was generated using the NEBNext Ultra II DNA library prep kit for Illumina (New England Biolabs, USA) following the manufacturer’s recommendations. We applied Fastp v0.19.5 (10) for quality filtering of the Illumina reads. Adapters were trimmed using Skewer v0.2.2 (11) (https://github.com/relipmoc/skewer). Quality checking of the Illumina reads was performed using FastQC v0.11.8 (https://www.bioinformatics.babraham.ac.uk/projects/fastqc/). Hybrid assemblies with the ONT and Illumina data were performed using Unicycler v0.4.8 (12) (https://github.com/rrwick/Unicycler/releases), and the genome sequences were checked for quality using QUAST v5.0.2 (13) (http://quast.sourceforge.net/). Complete circular DNA structures of the bacterial chromosomes and plasmids were automatically produced using Unicycler software. The genome sequences were submitted to the NCBI Prokaryotic Genome Annotation Pipeline (PGAP) v4.12 for annotation. Default parameters were used for all software unless otherwise specified.

All isolates contained plasmids, and we detected seven plasmids in one isolate (Table 1). From the whole-genome sequencing, the following antimicrobial resistance determinants were ascertained using ResFinder (14) (https://cge.cbs.dtu.dk/services/ResFinder/) and CARD (15) (https://card.mcmaster.ca/): *bla*_NDM-1_, *bla*_OXA-23_, *bla*_OXA-66_, *bla*_TEM1D_, *bla*_VEB-1_, *bla*_IMP-14_, *bla*_ADC-25_, *sul1*, *sul2*, *cmlA1*, *mphE*, *msrE*, *ARR2*, *tetB*, *tet*(39), *aac(3)*-*IId*, *aadA1*, *ant(2″)*-*Ia*, *aph(3′)*-*Ia*, *aph(3″)*-*Ib*, *aph(3′)*-*VI*, *aph(6)*-*Id*, and *armA*. The multilocus sequence type (MLST) findings using the PubMLST database indicated that two isolates were ST16 (or ST355), and the other isolates were ST2 (or ST195) and ST1479 (new sequence type), according to the Pasteur MLST scheme (with the Oxford MLST scheme in parentheses) (16, 17) (https://pubmlst.org/abaumannii/).

**TABLE 1:** Assembly metrics and accession numbers of four clinically extensively drug-resistant Acinetobacter baumannii strains

Strain	Sequence type	Total no. of bases (millions)	No. of reads (thousands)	*N*_50_ (bp)	BioSample accession no.	SRA accession no. for:		GenBank accession no.	Total no. of contigs	Type of contig	Total length (bp)	GC content (%)	No. of ORFs^*a*^	No. of rRNAs	No. of tRNAs
						ONT reads	Illumina reads								
A217	ST16	1,194	173	12,710	SAMN15596636	SRR12517409	SRR12517413	JACEIH000000000	5	Chromosome	3,897,448	39.05	3,606	18	75
										Plasmid	118,657	41.06	127	0	1
										Plasmid	78,040	43.88	82	0	0
		1,266	8,400	150											
										Plasmid	43,239	38.81	62	0	0
										Plasmid	12,366	34.46	14	0	0
A466	ST16	1,440	248	10,073	SAMN15596638	SRR12517407	SRR12517411	JACEIF000000000	2	Chromosome	3,944,195	39.02	3,683	18	74
		1,208	8,100	150						Plasmid	8,731	34.37	10	0	0
A522	ST2	1,481	222	12,765	SAMN15596639	SRR12517406	SRR12517410	JACEIJ000000000	4	Chromosome	3,897,674	39.05	3,621	18	75
										Plasmid	118,652	41.05	127	0	1
										Plasmid	66,679	43.99	69	0	0
		684	2,800	150											
										Plasmid	12,366	34.46	14	0	0
A423	ST1479	1,407	256	10,240	SAMN15596637	SRR12517408	SRR12517412	JACEIG000000000	8	Chromosome	3,806,348	39.00	3,517	18	75
										Plasmid	108,874	43.13	116	0	0
										Plasmid	95,364	41.49	104	0	1
										Plasmid	6,078	39.17	5	0	0
										Plasmid	4,554	41.26	4	0	0
		1,310	8,700	150											
										Plasmid	2,924	37.82	4	0	0
										Plasmid	2,816	38.74	4	0	0
										Plasmid	2,309	38.54	2	0	0

a ORFs, open reading frames.

### Data availability.

These sequences have been submitted to GenBank under BioProject accession number PRJNA647677. The accession numbers and assembly statistics are provided in Table 1.
